# Cold-Water Bathing

**Published:** 1860-12

**Authors:** 


### COLD-WATER BATHING.

A PROFESSIONAL gentleman of high character and great usefulness, writes: "My own experience is somewhat different from your advice on the subject of bathing. I have always found a warm bath debilitating, a cold one invigorating. When the thermometer was below zero, and sleeping in a room without fire, I have often, on rising, broken the ice in a vessel of water, and sponged myself all over before dressing, and I thought with decided benefit; and now, at sixty years of age, a cold shower-bath seems to inspire me with new life. You will think, perhaps, that I have very healthy lungs, and an extra supply of animal heat; on the contrary, I was given up in early life to die of consumption. I have been obliged to be very regular in my habits, and very careful of exposure. Some years ago, one of the most skillful practitioners in the city gave it as his opinion that a portion of one lung was entirely gone." On page 103 of the May JOURNAL a *per contra* case is given, of a cold-water bather who has since died. Single cases should not form rules. We advised bathing once a week in warm water, with soap and brush, as needful in summer-time for personal cleanliness; in winter, not so often. If such a bath "debilitates," it is most likely owing to its being too long continued. The whole operation might be performed in less than five minutes, and if ended with an instantaneous shower of cold water, such a bath could scarcely fail to invigorate. We do not advise a warm bath oftener than once a week. But we must consult nature and facts. Each man should bathe in a manner which, from observation and personal experiment, does him most good. In matters of health and disease, each must be his own rule. Immense mischief is daily done by ignoring this principle, which is at once the dictate of a sound philosophy and of common-sense.

Let not the reader run away with the impression that cold-water bathing cured this case of consumption, and that by its invigorating effects he is enabled to live in good health; for this restoration from an admitted consumptive condition, was owing to the fact that the course of the malady was changed, and its nature modified by an asthmatic turn of the disease, as for "twenty years and more, I was greatly afflicted with asthma." It is a settled fact in medicine, one of frequent record and of constant occurrence, that a consumptive who becomes an asthmatic, will with great certainty get well of his consumption, asthma being essentially and under all circumstances antagonistic of consumption. In consumption, a man can not get in enough air; in asthma, he can not get it out. In asthma, the lungs are too full of air; in consumption, not full enough. Being so full, distended by the confined air, that distension after a while becomes permanent, the asthma declines, leaving the lungs with a larger capability of receiving air than is natural; hence, although the lungs may have partly decayed away, those which remain, having greater capabilities, a man may have good health, who had actually lost a part of his lungs by consumption. It is precisely on these principles that we have treated consumptive cases, and with occasional success, for nearly twenty years; not generally succeeding, in consequence of the want of the moral courage of persistence on the part of the patient.

